# *N*-Ethyl-2,2-dimethyl-*N*-(3-methyl­phen­yl)propanamide

**DOI:** 10.1107/S1600536814001718

**Published:** 2014-01-29

**Authors:** B. S. Palakshamurthy, P. A. Suchetan, S Sreenivasa, N. K. Lokanath, T Madhu Chakrapani Rao

**Affiliations:** a Department of Studies and Research in Physics, U.C.S., Tumkur University, Tumkur, Karnataka 572 103, India; b Department of Studies and Research in Chemistry, U.C.S., Tumkur University, Tumkur, Karnataka 572 103, India; c Department of Studies and Research in Chemistry, Tumkur University, Tumkur, Karnataka 572 103, India; d Department of Studies in Physics, University of Mysore, Manasagangotri, Mysore, India; e Tadimety Aromatics Pvt Ltd, Hirehally Industrial Area, Tumkur, Karnataka 572 168, India

## Abstract

In the title compound, C_14_H_21_NO, the conformation across the N—C(O) bond is *syn*-periplanar, the C—N—C—C torsion being −5.9 (5)°. The atoms of the ethyl group attached to the N atom are disordered over two sets of sites with occupancy ratios of 0.65 (2):0.35 (2) (CH_2_) and 0.689 (14):0.311 (14) (CH_3_)are linked by very weak C—H⋯O inter­actions forming *C*(8) chains along [001]. C—H⋯π inter­actions link the mol­ecules along the *c-*axis direction.

## Related literature   

For hydrogen-bond motifs, see: Bernstein *et al.* (1995 ▶). For the biological activity of amides, see: Manojkumar *et al.* (2013*a* ▶,*b* ▶). Amide groups can provide structural rigidity to mol­ecules, see: Sreenivasa *et al.* (2013 ▶).
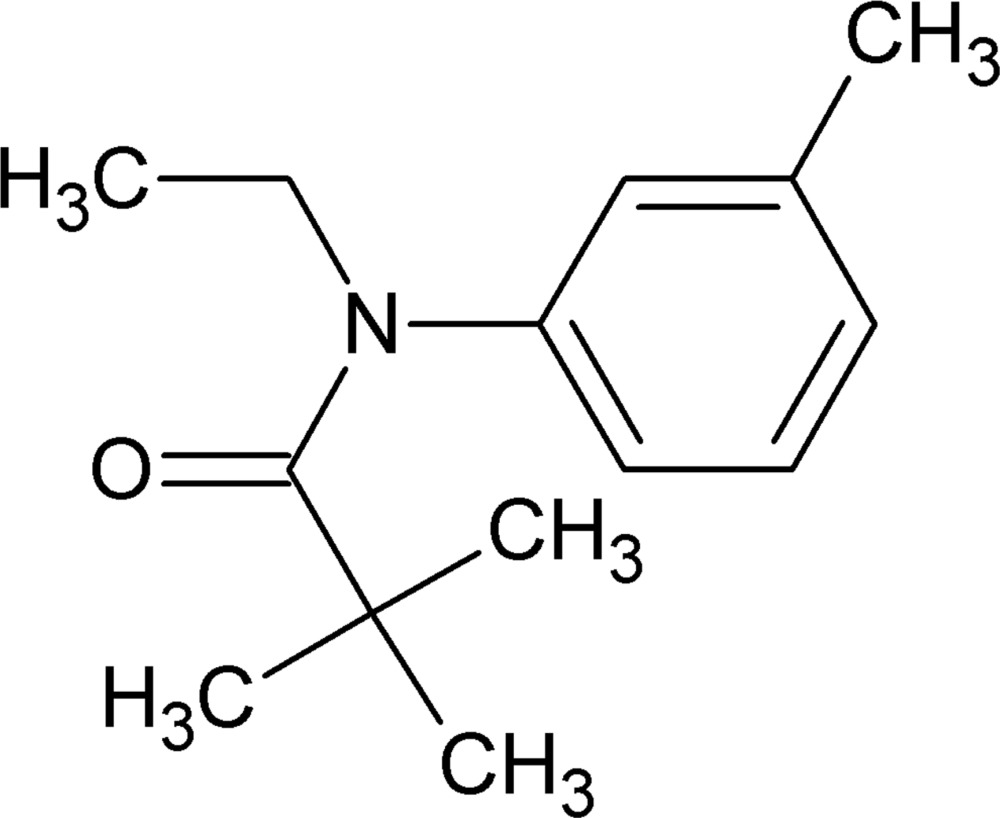


## Experimental   

### 

#### Crystal data  

C_14_H_21_NO
*M* *_r_* = 219.32
Monoclinic, 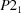

*a* = 7.631 (4) Å
*b* = 10.878 (7) Å
*c* = 8.350 (3) Å
β = 105.60 (2)°
*V* = 667.6 (6) Å^3^
*Z* = 2
Cu *K*α radiation
μ = 0.52 mm^−1^
*T* = 294 K
0.22 × 0.20 × 0.16 mm

#### Data collection  

Bruker APEXII diffractometer
Absorption correction: multi-scan (*SADABS*; Bruker, 2009 ▶) *T*_min_ = 0.893, *T*_max_ = 0.921
3786 measured reflections
2016 independent reflections
1883 reflections with *I* > 2σ(*I*)
*R*_int_ = 0.034

#### Refinement  

*R*[*F* ^2^ > 2σ(*F* ^2^)] = 0.057
*wR*(*F* ^2^) = 0.160
*S* = 1.06
2016 reflections
172 parameters
55 restraints
H-atom parameters constrained
Δρ_max_ = 0.34 e Å^−3^
Δρ_min_ = −0.16 e Å^−3^

### 

Data collection: *APEX2* (Bruker, 2009 ▶); cell refinement: *APEX2* and *SAINT-Plus* (Bruker, 2009 ▶); data reduction: *SAINT-Plus* and *XPREP* (Bruker, 2009 ▶); program(s) used to solve structure: *SHELXS97* (Sheldrick, 2008 ▶); program(s) used to refine structure: *SHELXL97* (Sheldrick, 2008 ▶); molecular graphics: *Mercury* (Macrae *et al.*, 2008 ▶); software used to prepare material for publication: *SHELXL97* (Sheldrick, 2008 ▶).

## Supplementary Material

Crystal structure: contains datablock(s) I. DOI: 10.1107/S1600536814001718/hg5371sup1.cif

Structure factors: contains datablock(s) I. DOI: 10.1107/S1600536814001718/hg5371Isup2.hkl

Supporting information file. DOI: 10.1107/S1600536814001718/hg5371Isup3.cml

CCDC reference:

Additional supporting information:  crystallographic information; 3D view; checkCIF report

## Figures and Tables

**Table 1:** Hydrogen-bond geometry (Å, °) *Cg* is the centoid of the benzene ring.

*D*—H⋯*A*	*D*—H	H⋯*A*	*D*⋯*A*	*D*—H⋯*A*
C1—H1⋯O^i^	0.93	2.62	3.481 (2)	153
C14—H14*A*⋯*Cg* ^ii^	0.96	2.85	3.769 (8)	161

Symmetry codes: (i) 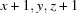; (ii) 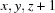.

**Fig. 1.**
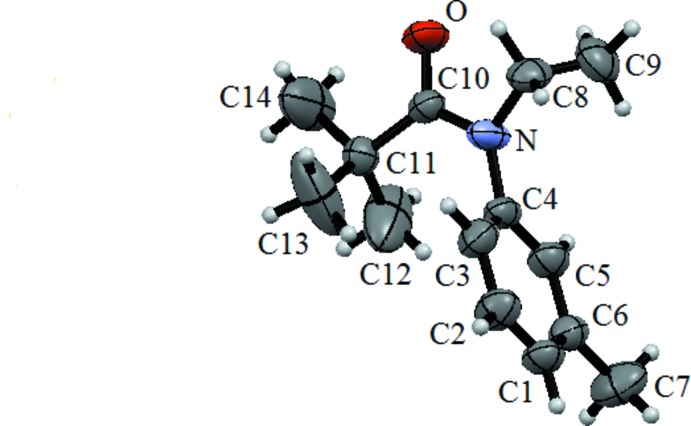
Molecular structure of the title compound, showing displacement ellipsoids drawn at the 50% probability level. Only the major components of the disordered atoms are shown.

**Fig. 2.**
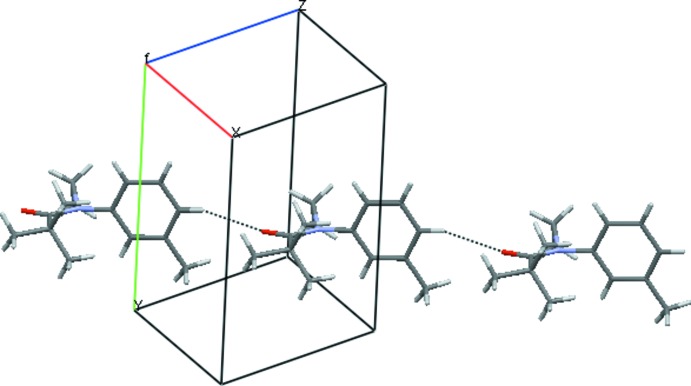
Molecular packing foming C(8) chains with hydrogen bonding shown as dashed lines.

**Fig. 3.**
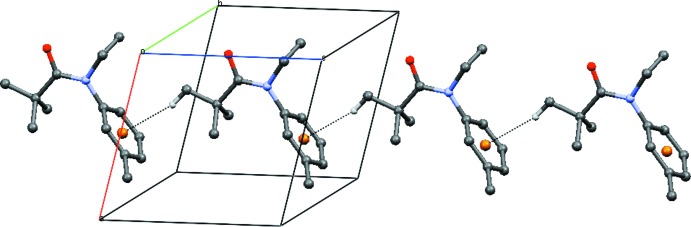
Stacking of molecules along c axis through C—H···Cg interactions. Cg is the centroid of the benzene ring. H-atoms not involved in H-bonding are ommitted for clarity.

## supplementary crystallographic information

### 1. Comment

Amides are very common in nature, formed easily and provides structural rigidity to the molecules (Sreenivasa *et al.* 2013). Amides show a broad spectrum of pharmacological properties, including antibacterial (Manojkumar *et al.* 2013*a*), anti-inflammatory, antioxidant, analgesic and antiviral activity (Manojkumar *et al.* 2013*b*). Keeping this in mind, the crystal structure of the title compound was determined.

### 2. Experimental

N-Ethyl-3-methylaniline (1.00g,7.4 mmol) was taken in dry dichloromethane (10 mL) and the solution was cooled to 0 ^o^C. To this reaction mixture 2,2-dimethylpropanoyl chloride (0.888 g, 7.4 mmol) in dichloromethane and triethylamine (1.49g, 1.48 mmol) were added slowly and the mixture was heated to 50^o^C for 4 hours. Reaction was monitored by TLC. Reaction mixture was cooled and washed with 10% NaHCO_3_ solution. The organic layer was separated, dried and concentrated to obtained crude product which was purified by column chromatography using petroleum ether: ethyl acetate (7:3) as eluent. Yellow prisms of the title compound were obtained from slow evapouration of the solution of the compound in petroleum ether: ethyl acetate (7:3).

### 3. Refinement

The H atoms were positioned with idealized geometry using a riding model with C-H = 0.93-0.96Å. All H atoms were refined with isotropic displacement parameters (set to 1.2-1.5 times of the Ueq of the parent atom). Flack parameter value (Flack, 1983) of 0.5 (5) was obtained in the final structure factor calculation, the presence of pseudosymmetry can lead to uncertainties about the correct space group, especially in the presence of twinning.

The C8 and C9 atoms of the ethyl group attached to N atom are disordered with site occupation factors of 0.65 (2):0.35 (2) and 0.689 (14):0.311 (14) respectively.

### Crystal data

**Table:**

C_14_H_21_NO	Prism
*M**_r_* = 219.32	*D*_x_ = 1.091 Mg m^−^^3^
Monoclinic, *P*2_1_	Melting point: 492 K
Hall symbol: P 2yb	Cu *K*α radiation, λ = 1.54178 Å
*a* = 7.631 (4) Å	Cell parameters from 172 reflections
*b* = 10.878 (7) Å	θ = 5.5–65.5°
*c* = 8.350 (3) Å	µ = 0.52 mm^−^^1^
β = 105.60 (2)°	*T* = 294 K
*V* = 667.6 (6) Å^3^	Prism, yellow
*Z* = 2	0.22 × 0.20 × 0.16 mm
*F*(000) = 240	

### Data collection

**Table:**

Bruker APEXII diffractometer	1883 reflections with *I* > 2σ(*I*)
Radiation source: fine-focus sealed tube	*R*_int_ = 0.034
Graphite monochromator	θ_max_ = 65.5°, θ_min_ = 5.5°
phi and ω scans	*h* = −8→8
Absorption correction: multi-scan (*SADABS*; Bruker, 2009)	*k* = −11→12
*T*_min_ = 0.893, *T*_max_ = 0.921	*l* = −9→9
3786 measured reflections	1012 standard reflections every 2 reflections
2016 independent reflections	intensity decay: 1%

### Refinement

**Table:**

Refinement on *F*^2^	Secondary atom site location: difference Fourier map
Least-squares matrix: full	Hydrogen site location: inferred from neighbouring sites
*R*[*F*^2^ > 2σ(*F*^2^)] = 0.057	H-atom parameters constrained
*wR*(*F*^2^) = 0.160	*w* = 1/[σ^2^(*F*_o_^2^) + (0.093*P*)^2^ + 0.1495*P*] where *P* = (*F*_o_^2^ + 2*F*_c_^2^)/3
*S* = 1.06	(Δ/σ)_max_ < 0.001
2016 reflections	Δρ_max_ = 0.34 e Å^−^^3^
172 parameters	Δρ_min_ = −0.16 e Å^−^^3^
55 restraints	Extinction correction: *SHELXL97* (Sheldrick, 2008), Fc^*^=kFc[1+0.001xFc^2^λ^3^/sin(2θ)]^-1/4^
0 constraints	Extinction coefficient: 0.018 (4)
Primary atom site location: structure-invariant direct methods	

### Special details

**Table:**

Geometry. All e.s.d.'s (except the e.s.d. in the dihedral angle between two l.s. planes) are estimated using the full covariance matrix. The cell e.s.d.'s are taken into account individually in the estimation of e.s.d.'s in distances, angles and torsion angles; correlations between e.s.d.'s in cell parameters are only used when they are defined by crystal symmetry. An approximate (isotropic) treatment of cell e.s.d.'s is used for estimating e.s.d.'s involving l.s. planes.
Refinement. Refinement of *F*^2^ against ALL reflections. The weighted *R*-factor *wR* and goodness of fit *S* are based on *F*^2^, conventional *R*-factors *R* are based on *F*, with *F* set to zero for negative *F*^2^. The threshold expression of *F*^2^ > σ(*F*^2^) is used only for calculating *R*-factors(gt) *etc*. and is not relevant to the choice of reflections for refinement. *R*-factors based on *F*^2^ are statistically about twice as large as those based on *F*, and *R*- factors based on ALL data will be even larger.

### Fractional atomic coordinates and isotropic or equivalent isotropic displacement parameters (Å^2^)

**Table:**

	*x*	*y*	*z*	*U*_iso_*/*U*_eq_	Occ. (<1)
O	0.2284 (2)	0.7636 (3)	0.6964 (2)	0.0725 (7)	
N	0.4145 (3)	0.7530 (3)	0.9461 (2)	0.0670 (8)	
C11	0.5373 (3)	0.7418 (3)	0.6942 (3)	0.0489 (6)	
C6	0.8300 (4)	0.8185 (3)	1.2843 (3)	0.0494 (6)	
C10	0.3855 (3)	0.7522 (3)	0.7806 (3)	0.0452 (6)	
C4	0.5820 (3)	0.7342 (3)	1.0730 (3)	0.0487 (7)	
C5	0.6765 (4)	0.8340 (3)	1.1519 (3)	0.0520 (7)	
H5	0.6374	0.9128	1.1164	0.062*	
C3	0.6371 (4)	0.6191 (3)	1.1298 (3)	0.0586 (8)	
H3	0.5716	0.5510	1.0789	0.070*	
C2	0.7875 (4)	0.6028 (3)	1.2607 (4)	0.0602 (8)	
H2	0.8249	0.5239	1.2975	0.072*	
C1	0.8829 (4)	0.7029 (3)	1.3376 (3)	0.0520 (7)	
H1	0.9848	0.6916	1.4271	0.062*	
C7	0.9325 (5)	0.9283 (4)	1.3690 (5)	0.0834 (11)	
H7A	0.9820	0.9109	1.4851	0.125*	
H7B	0.8515	0.9974	1.3563	0.125*	
H7C	1.0295	0.9472	1.3200	0.125*	
C14	0.4518 (6)	0.7552 (8)	0.5111 (5)	0.129 (2)	
H14A	0.5454	0.7608	0.4542	0.193*	
H14B	0.3787	0.8283	0.4908	0.193*	
H14C	0.3766	0.6849	0.4710	0.193*	
C13	0.6307 (11)	0.6209 (5)	0.7262 (9)	0.136 (3)	
H13A	0.5417	0.5564	0.7079	0.205*	
H13B	0.7044	0.6178	0.8392	0.205*	
H13C	0.7066	0.6103	0.6523	0.205*	
C12	0.6785 (8)	0.8393 (6)	0.7487 (7)	0.135 (3)	
H12A	0.7324	0.8325	0.8663	0.202*	
H12B	0.6230	0.9187	0.7237	0.202*	
H12C	0.7708	0.8293	0.6910	0.202*	
C8A	0.2598 (9)	0.7879 (9)	1.0135 (7)	0.052 (2)	0.65 (2)
H8A1	0.1744	0.8395	0.9348	0.063*	0.65 (2)
H8A2	0.3035	0.8329	1.1169	0.063*	0.65 (2)
C8B	0.2459 (15)	0.7104 (19)	1.0046 (14)	0.059 (4)	0.35 (2)
H8B1	0.1529	0.6742	0.9142	0.070*	0.35 (2)
H8B2	0.2801	0.6522	1.0958	0.070*	0.35 (2)
C9A	0.1691 (10)	0.6700 (7)	1.0428 (8)	0.087 (2)	0.689 (14)
H9A1	0.1289	0.6256	0.9400	0.131*	0.689 (14)
H9A2	0.0664	0.6887	1.0843	0.131*	0.689 (14)
H9A3	0.2542	0.6207	1.1226	0.131*	0.689 (14)
C9B	0.1877 (19)	0.8243 (14)	1.0567 (15)	0.071 (4)	0.311 (14)
H9B1	0.2883	0.8636	1.1337	0.107*	0.311 (14)
H9B2	0.0925	0.8096	1.1096	0.107*	0.311 (14)
H9B3	0.1429	0.8765	0.9617	0.107*	0.311 (14)

### Atomic displacement parameters (Å^2^)

**Table:**

	*U*^11^	*U*^22^	*U*^33^	*U*^12^	*U*^13^	*U*^23^
O	0.0395 (10)	0.130 (2)	0.0405 (9)	0.0096 (12)	−0.0029 (8)	−0.0003 (11)
N	0.0294 (10)	0.136 (2)	0.0338 (10)	0.0065 (14)	0.0055 (8)	0.0032 (14)
C11	0.0481 (13)	0.0608 (15)	0.0394 (12)	0.0028 (13)	0.0147 (10)	0.0013 (11)
C6	0.0429 (14)	0.0613 (17)	0.0417 (12)	−0.0053 (13)	0.0072 (10)	0.0004 (12)
C10	0.0375 (12)	0.0603 (14)	0.0343 (11)	−0.0002 (12)	0.0038 (9)	−0.0004 (11)
C4	0.0336 (12)	0.0806 (19)	0.0298 (10)	0.0034 (13)	0.0051 (9)	0.0036 (12)
C5	0.0456 (15)	0.0645 (17)	0.0427 (13)	0.0073 (13)	0.0063 (11)	0.0077 (12)
C3	0.0559 (17)	0.0689 (19)	0.0469 (15)	−0.0075 (15)	0.0067 (13)	−0.0085 (13)
C2	0.0631 (18)	0.0565 (17)	0.0542 (15)	0.0061 (14)	0.0040 (14)	0.0069 (13)
C1	0.0417 (15)	0.0677 (18)	0.0404 (12)	0.0050 (13)	0.0003 (11)	0.0069 (12)
C7	0.079 (2)	0.073 (2)	0.083 (3)	−0.0182 (19)	−0.0049 (19)	−0.0069 (18)
C14	0.086 (3)	0.254 (7)	0.0529 (18)	0.027 (4)	0.0323 (19)	0.021 (3)
C13	0.189 (6)	0.118 (4)	0.150 (5)	0.083 (4)	0.126 (5)	0.052 (3)
C12	0.134 (4)	0.178 (5)	0.125 (4)	−0.087 (4)	0.091 (4)	−0.062 (4)
C8A	0.038 (3)	0.075 (5)	0.045 (2)	0.006 (3)	0.0115 (19)	−0.008 (3)
C8B	0.037 (5)	0.083 (10)	0.054 (5)	−0.014 (6)	0.009 (4)	0.001 (5)
C9A	0.066 (4)	0.110 (5)	0.101 (4)	−0.013 (3)	0.049 (3)	0.001 (4)
C9B	0.057 (7)	0.095 (8)	0.073 (7)	0.006 (6)	0.035 (5)	0.003 (6)

### Geometric parameters (Å, º)

**Table:**

O—C10	1.222 (3)	C7—H7C	0.9600
N—C10	1.339 (3)	C14—H14A	0.9600
N—C4	1.439 (3)	C14—H14B	0.9600
N—C8A	1.488 (7)	C14—H14C	0.9600
N—C8B	1.564 (12)	C13—H13A	0.9600
C11—C13	1.486 (5)	C13—H13B	0.9600
C11—C12	1.493 (5)	C13—H13C	0.9600
C11—C14	1.499 (5)	C12—H12A	0.9600
C11—C10	1.525 (3)	C12—H12B	0.9600
C6—C1	1.359 (4)	C12—H12C	0.9600
C6—C5	1.388 (4)	C8A—C9A	1.508 (13)
C6—C7	1.497 (5)	C8A—H8A1	0.9700
C4—C3	1.364 (4)	C8A—H8A2	0.9700
C4—C5	1.370 (4)	C8B—C9B	1.42 (3)
C5—H5	0.9300	C8B—H8B1	0.9700
C3—C2	1.367 (4)	C8B—H8B2	0.9700
C3—H3	0.9300	C9A—H9A1	0.9600
C2—C1	1.370 (4)	C9A—H9A2	0.9600
C2—H2	0.9300	C9A—H9A3	0.9600
C1—H1	0.9300	C9B—H9B1	0.9600
C7—H7A	0.9600	C9B—H9B2	0.9600
C7—H7B	0.9600	C9B—H9B3	0.9600
			
C10—N—C4	128.83 (19)	H7B—C7—H7C	109.5
C10—N—C8A	117.7 (3)	C11—C14—H14A	109.5
C4—N—C8A	113.3 (2)	C11—C14—H14B	109.5
C10—N—C8B	113.5 (5)	H14A—C14—H14B	109.5
C4—N—C8B	111.7 (4)	C11—C14—H14C	109.5
C13—C11—C12	107.6 (5)	H14A—C14—H14C	109.5
C13—C11—C14	109.0 (4)	H14B—C14—H14C	109.5
C12—C11—C14	108.8 (4)	C11—C13—H13A	109.5
C13—C11—C10	111.7 (3)	C11—C13—H13B	109.5
C12—C11—C10	112.4 (3)	H13A—C13—H13B	109.5
C14—C11—C10	107.3 (2)	C11—C13—H13C	109.5
C1—C6—C5	119.1 (3)	H13A—C13—H13C	109.5
C1—C6—C7	120.8 (3)	H13B—C13—H13C	109.5
C5—C6—C7	120.1 (3)	C11—C12—H12A	109.5
O—C10—N	117.2 (2)	C11—C12—H12B	109.5
O—C10—C11	119.2 (2)	H12A—C12—H12B	109.5
N—C10—C11	123.6 (2)	C11—C12—H12C	109.5
C3—C4—C5	119.1 (2)	H12A—C12—H12C	109.5
C3—C4—N	121.1 (3)	H12B—C12—H12C	109.5
C5—C4—N	119.3 (3)	N—C8A—C9A	106.8 (6)
C4—C5—C6	120.6 (3)	N—C8A—H8A1	110.4
C4—C5—H5	119.7	C9A—C8A—H8A1	110.4
C6—C5—H5	119.7	N—C8A—H8A2	110.4
C4—C3—C2	120.7 (3)	C9A—C8A—H8A2	110.4
C4—C3—H3	119.6	H8A1—C8A—H8A2	108.6
C2—C3—H3	119.6	C9B—C8B—N	100.9 (13)
C3—C2—C1	119.9 (3)	C9B—C8B—H8B1	111.6
C3—C2—H2	120.1	N—C8B—H8B1	111.6
C1—C2—H2	120.1	C9B—C8B—H8B2	111.6
C6—C1—C2	120.6 (2)	N—C8B—H8B2	111.6
C6—C1—H1	119.7	H8B1—C8B—H8B2	109.4
C2—C1—H1	119.7	C8A—C9A—H9A1	109.5
C6—C7—H7A	109.5	C8A—C9A—H9A2	109.5
C6—C7—H7B	109.5	H9A1—C9A—H9A2	109.5
H7A—C7—H7B	109.5	C8A—C9A—H9A3	109.5
C6—C7—H7C	109.5	H9A1—C9A—H9A3	109.5
H7A—C7—H7C	109.5	H9A2—C9A—H9A3	109.5
			
C4—N—C10—O	175.9 (3)	C8B—N—C4—C5	−109.8 (9)
C8A—N—C10—O	−9.8 (6)	C3—C4—C5—C6	2.2 (4)
C8B—N—C10—O	25.7 (9)	N—C4—C5—C6	174.5 (2)
C4—N—C10—C11	−5.9 (5)	C1—C6—C5—C4	−1.7 (4)
C8A—N—C10—C11	168.3 (5)	C7—C6—C5—C4	179.8 (3)
C8B—N—C10—C11	−156.1 (9)	C5—C4—C3—C2	−1.8 (4)
C13—C11—C10—O	−116.5 (5)	N—C4—C3—C2	−174.0 (3)
C12—C11—C10—O	122.5 (4)	C4—C3—C2—C1	0.9 (5)
C14—C11—C10—O	2.9 (5)	C5—C6—C1—C2	0.8 (4)
C13—C11—C10—N	65.3 (5)	C7—C6—C1—C2	179.3 (3)
C12—C11—C10—N	−55.7 (5)	C3—C2—C1—C6	−0.4 (4)
C14—C11—C10—N	−175.2 (4)	C10—N—C8A—C9A	94.8 (5)
C10—N—C4—C3	−88.2 (4)	C4—N—C8A—C9A	−90.0 (5)
C8A—N—C4—C3	97.3 (5)	C8B—N—C8A—C9A	4.1 (8)
C8B—N—C4—C3	62.4 (9)	C10—N—C8B—C9B	−107.2 (7)
C10—N—C4—C5	99.6 (4)	C4—N—C8B—C9B	97.4 (8)
C8A—N—C4—C5	−74.9 (5)	C8A—N—C8B—C9B	−2.2 (6)

### Hydrogen-bond geometry (Å, º)

Cg is the centoid of the benzene ring.

**Table:**

*D*—H···*A*	*D*—H	H···*A*	*D*···*A*	*D*—H···*A*
C1—H1···O^i^	0.93	2.62	3.481 (2)	153
C14—H14*A*···*Cg*^ii^	0.96	2.85	3.769 (8)	161

Symmetry codes: (i) *x*+1, *y*, *z*+1; (ii) *x*, *y*, *z*+1.
